# Impact of Intervention Measures on MRSA Clonal Type and Carriage Site Prevalence

**DOI:** 10.1128/mBio.00218-16

**Published:** 2016-03-08

**Authors:** Marco Cassone, Sara E. McNamara, Mary Beth Perri, Marcus Zervos, Lona Mody

**Affiliations:** a Division of Geriatric and Palliative Care Medicine, University of Michigan, Ann Arbor, Michigan, USA; b Division of Infectious Diseases, Henry Ford Health System, Detroit, Michigan, USA; c Geriatrics Research Education and Clinical Center, VA Ann Arbor Healthcare System, Ann Arbor, Michigan, USA

## LETTER

We read with great interest the article by Senn and others (1) as a prime example of how detailed molecular analysis can unravel the epidemiological patterns that methicillin-resistant *Staphylococcus aureus* (MRSA) strains follow in the colonizing process and the usefulness of extranasal sampling to uncover MRSA prevalence and spreading pathways.

Screening of MRSA on multiple body sites is well known to greatly enhance isolation sensitivity. However, the study by Senn et al. is among the first to open a new awareness about qualitative differences in the behavior of specific MRSA strains. Based on our experience, we concur with the implied author’s conclusion that screening protocols limited to the nares may lead to selective under- or overestimation of the prevalence of specific strains, with the potential of greatly undermining surveillance efforts. As in Senn’s study, our data also paint a picture of variability in strain behavior, which in our case manifests itself in the response to interventions.

We performed molecular typing of 471 confirmed MRSA strains isolated during our targeted infection prevention (TIP) clinical trial (2), which focused on high-risk nursing home residents with an indwelling urinary catheter or feeding tube. The intervention program included use of preemptive barrier precautions, deidentified feedback, an interactive hand hygiene program, and an interactive infection prevention educational program for all health care workers (3). Strains were assigned to 1 of 18 groups based on SmaI pulsed-field gel electrophoresis (PFGE), Panton-Valentine leukocidin (PVL) cytotoxin PCR (4), *agr* typing (5–7), or SCC*mec* typing (8–10) and were stratified by site of isolation (nasal, oral, groin, perianal, feeding tube insertion site, urinary catheter insertion site, and wound). We observed significant changes in the ratio of nasal to extranasal colonization in the intervention facilities compared to the control facilities, among specific molecular types (Table 1). The ratio of nasal to extranasal sample positivity was fairly consistent among different molecular types in the control group. In the intervention group, however, the ratio was significantly decreased for some strains and increased for others. In the absence of molecular typing, those changes would have been unnoticed. Moreover, the total number of isolates from all sites also showed different reduction percentages with the intervention: type 2 isolates, for example, decreased by 65%, and type 3 did not decrease at all. Type 2 isolates represent PFGE USA300 PVL^+^ strains, which are commonly community acquired. Most USA300 isolates described so far have been recovered from wounds and skin and soft tissue infections, where they can be the predominant type (11). However, in nursing homes, USA300 isolates have been uncommon until recently (12). As in Senn and colleagues’ observations, differential behavior of selected strains was inferred from screening extranasal sites and molecular typing. In our case, this phenomenon manifested as a different response to interventions. This observation has few precedents in the literature, except for specific resistance to topical antiseptics (13, 14).

**TABLE 1:** Distribution of the most represented MRSA types (20 or more isolates) for nasal versus extranasal colonization in control and intervention facilities

Type^a^	PFGE profile	No. of control isolates (3,258 swabs tested)				No. of isolates with intervention (3,149 swabs tested)			
		Site of isolation		Total	Extranasal/nasal ratio	Site of isolation		Total	Extranasal/nasal ratio
		Nasal	Extranasal			Nasal	Extranasal		
1	USA100	36	96	132	2.7	35	59	94^b^	1.7^b^
2	USA300 PVL^+^	16	29	45	1.8	1	15	16^b^	15^b^
3	USA non-100-1100	16	29	45	1.8	8	41	49	5.1^b^
All		88	182	270	2.1	57	144	201^b^	2.5

a Type grouping was assigned based on 18 unique combinations of PFGE (1% tolerance), *agr* typing, SCC*mec* typing, or PVL typing.

b *P* values of <0.05 (intervention vs. control).

During the 3-year study period, we did not observe outbreaks in any of the 12 facilities. This study, conducted in the nursing home setting, adds confirmation that MRSA strain-specific characteristics influences anatomic site colonization patterns and the response to interventions. This must be taken into account when designing resource-intensive interventions aimed at decreasing MRSA burden.
